# The Roles of Superoxide on At-Level Spinal Cord Injury Pain in Rats

**DOI:** 10.3390/ijms22052672

**Published:** 2021-03-06

**Authors:** Bong Hyo Lee, Jonghoon Kang, Hee Young Kim, Young S. Gwak

**Affiliations:** 1 Department of Acupuncture, Moxibustion and Acupoint, College of Korean Medicine, Daegu Haany University, Daegu 42158, Korea; dlqhdgy@dhu.ac.kr; 2 Research Center for Herbal Convergence on Liver Disease, Daegu Haany University, Daegu 42158, Korea; hykim@dhu.ac.kr; 3 Department of Biology, Valdosta State University, Valdosta, GA 31698, USA; jkang@valdosta.edu; 4 Department of Physiology, College of Korean Medicine, Daegu Haany University, Daegu 42158, Korea

**Keywords:** at-level, CamKII, ionotropic glutamate receptors, neuropathic pain, reactive oxygen species, spinal cord injury

## Abstract

Background: In the present study, we examined superoxide-mediated excitatory nociceptive transmission on at-level neuropathic pain following spinal thoracic 10 contusion injury (SCI) in male Sprague Dawley rats. Methods: Mechanical sensitivity at body trunk, neuronal firing activity, and expression of superoxide marker/ionotropic glutamate receptors (iGluRs)/CamKII were measured in the T7/8 dorsal horn, respectively. Results: Topical treatment of superoxide donor t-BOOH (0.4 mg/kg) increased neuronal firing rates and pCamKII expression in the naïve group, whereas superoxide scavenger Tempol (1 mg/kg) and non-specific ROS scavenger PBN (3 mg/kg) decreased firing rates in the SCI group (* *p* < 0.05). SCI showed increases of iGluRs-mediated neuronal firing rates and pCamKII expression (* *p* < 0.05); however, t-BOOH treatment did not show significant changes in the naïve group. The mechanical sensitivity at the body trunk in the SCI group (6.2 ± 0.5) was attenuated by CamKII inhibitor KN-93 (50 μg, 3.9 ± 0.4) or Tempol (1 mg, 4 ± 0.4) treatment (* *p* < 0.05). In addition, the level of superoxide marker Dhet showed significant increase in SCI rats compared to the sham group (11.7 ± 1.7 vs. 6.6 ± 1.5, * *p* < 0.05). Conclusions: Superoxide and the pCamKII pathway contribute to chronic at-level neuropathic pain without involvement of iGluRs following SCI.

## 1. Introduction

Reactive oxygen species (ROS) are important substrates for excitatory nociceptive transmission in the spinal dorsal horn [1,2]. ROS are generally produced by cellular metabolism, and the level of intracellular ROS is strictly controlled by the redox cycle via pro-oxidant and ROS scavengers [3,4,5]. However, neurotrauma, such as spinal cord injury (SCI), often results in the overproduction of ROS in the spinal dorsal horn followed by membrane and cellular physiological changes [6,7,8]. 

Superoxide is the major ROS in the nervous system and readily converts to hydroxyl radical, which is a highly reactive ROS involved in neuronal damages in SCI rats [6,7,9]. We previously showed that inhibiting superoxide overproduction with Tempol (4-hydroxy-TEMPO, 4-hydroxy-2,2,6,6-tetramethylpiperidine-1-oxyl) significantly attenuated SCI-induced below-level neuropathic pain and neuronal hyperexcitability in the lumbar spinal dorsal horn [10], suggesting that superoxide plays a key role in excitatory nociceptive transmission in the spinal dorsal horn after SCI. We and others have consistently showed that excitatory synaptic transmission facilitates the development and maintenance of neuropathic pain [11,12]. For example, the activation of ionotropic glutamate receptors, such as α-amino-3-hydroxy-5-methyl-4-isoxazolepropionic acid (AMPA) and N-methyl-D-aspartate (NMDA) is associated with neuropathic pain after neural damage [13,14,15]. Mechanistically, SCI activates glutamate receptors and the mitogen-activated protein kinase (MAPK) family-mediated intracellular downstream pathway in neuropathic pain conditions following SCI [15,16]. SCI also induces overproduction of ROS that significantly contribute to intracellular activation of the calcium-dependent protein kinase Ca^2+^/calmodulin-dependent protein kinase II (pCamKII), which contributes to neuropathic pain and spinal dorsal horn neuronal hyperexcitability in a remote region (below-level) following SCI [10]. Taken together, these results suggest that the potential coupling of ROS and intracellular calcium-dependent signaling contribute to neuropathic pain following SCI. However, little is known about the role of superoxide in the membrane-bound receptor and intracellular nociceptive pathway in the context of at-level neuropathic pain following SCI. 

Therefore, in the present study, we examined superoxide-mediated activation of the nociceptive pathway in a rat model of at-level neuropathic pain and neuronal hyperexcitability following thoracic contusion injury.

## 2. Results

### 2.1. Contribution of ROS to Neuronal Firing 

**Figure 1:**
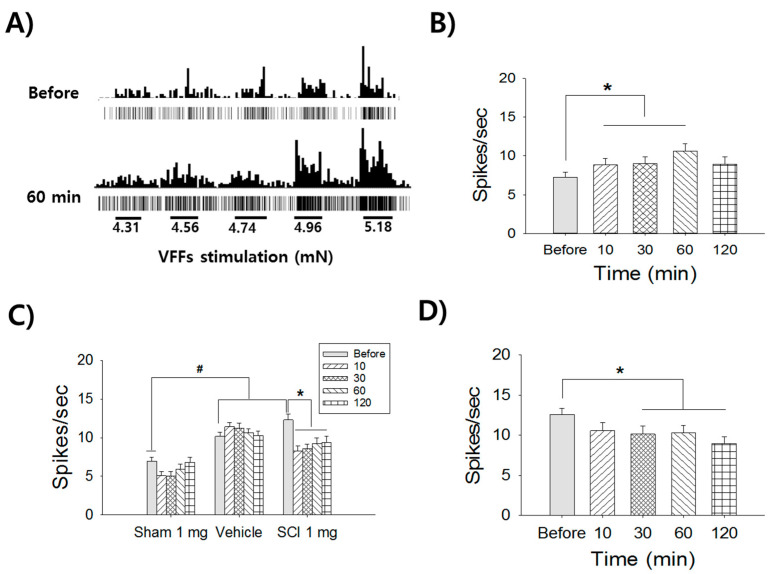
The contribution of superoxide on thoracic WDR neuronal firing rates. (**A**) The single WDR neuronal firing rates show increasing patterns to 5 different graded-intensity stimuli of VFFs prior to t-BOOH treatment (Before) and after (60 min) in naïve rats. Each VFF stimulation was applied for 10 s at the dorsal body trunk. (**B**) Cumulated firing rates show significant increases up to 60 min in the naïve group. (**C**) Prior to drug application, SCI-induced neuronal firing rates (Before) show significant increases in both the vehicle and SCI 1mg groups compared to the sham control (^#^ *p* < 0.05). Application of Tempol 1 mg significantly attenuates WDR neuronal firing rates from 10 min to 120 min, whereas the vehicle shows no changes in SCI rats (* *p* < 0.05). (**D**) Application of PBN 3 mg significantly attenuates WDR neuronal firing rates from 30 min to 120 min (* *p* < 0.05).

To determine whether superoxide contributes to thoracic dorsal horn neuronal hyperexcitability in naïve rats, we analyzed the firing rates of thoracic 7/8 wide dynamic range (T7/8 WDR) neurons in response to mechanical stimulation applied at the body trunk. After intrathecal administration of t-butylhydroperoxide (t-BOOH, 0.4 mg/kg), WDR neurons increased their firing rates in response to von Frey filament (VFF) stimuli (Figure 1A). One hour after treatment, the firing rate of the treated group was 10.6 ± 0.9 spikes/s, representing a significant increase compared to that before treatment (7.2 ± 0.6 spikes/s), as measured in 14 neurons in five rats (*p* = 0.036, Figure 1B). To determine whether SCI-induced overproduction of ROS contributed to WDR neuronal hyperexcitability, the firing rates of WDR neurons in response to VFF stimuli were assessed in sham (11 neurons/4 rats), SCI + vehicle (13 neurons/13 rats), SCI + Tempol (8 neurons/8 rats, 1 mg/kg), and SCI + N-tert-butyl-α-phenylnitrone (PBN, 8 neurons/8 rats, 3 mg/kg) rats. The firing rate of WDR neurons in the sham group was 6.9 ± 0.5 spikes/s, which was significantly different than that of the SCI + vehicle group (10.2 ± 0.6 spikes/s, *p* = 0.018) and SCI + Tempol group (12.3 ± 0.7 spikes/s, *p* = 0.008). However, 10 min after administering Tempol to the SCI + Tempol group, the firing rate decreased significantly to 8.2 ± 0.7 spikes/s (*p* = 0.006), and this decrease persisted for more than 1 hour, whereas the SCI + vehicle group did not change significantly (Figure 1C). In addition, PBN administration reduced the firing rate 30 min after administration, and this effect persisted for more than 1 hour (*p* = 0.016, Figure 1D). 

### 2.2. ROS is not Associated with Ionotropic Glutamate Receptor Expression

**Figure 2:**
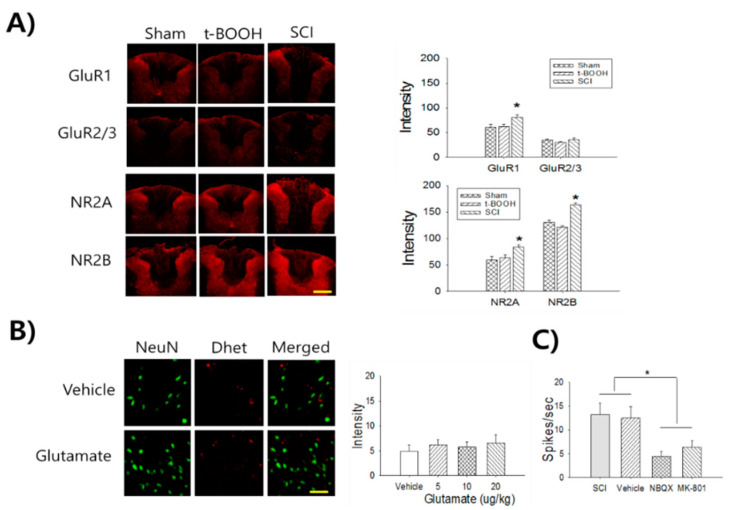
The contribution of ionotropic glutamate receptors on WDR neuronal firing rates after SCI. (**A**) The treatment of t-BOOH does not show significant changes in the expression of AMPA (GluR1 and GluR2/3) and NMDA (NR2A and NR2B) receptors, whereas the SCI group shows significant increases of GluR1, NR2A, and NR2B expression. Scale bar: 300 μm. (**B**) The treatment of glutamate (20 ug) does not show significant changes of superoxide production. Scale bar: 50 μm. (**C**) The treatment of NBQX (1 μg) and MK801 (50 μg) significantly attenuate WDR neuronal firing rates compared to SCI and vehicle groups (* *p* < 0.05).

To evaluate superoxide-mediated nociceptive signaling, we compared t-BOOH- and SCI-mediated ionotropic glutamate receptor and pCamKII expression in spinal dorsal horn neurons at thoracic level 7/8 (T7/8). One hour after 0.4 mg/kg intrathecal t-BOOH (n = 8 rats), the expression of AMAP receptors (GluR1 and GluR2/3) and NMDA receptors (NR2A and NR2B) was not different (Figure 2A). However, the expression of GluR1 (*p* = 0.04), NR2A (*p* = 0.033), and NR2B (*p* = 0.018) increased significantly after SCI (Figure 2A). In addition, intrathecal administration of glutamate (nociceptive inducible dose, 20 μg/n = 5) did not affect superoxide production (Figure 2B). AMPA and NMDA receptor antagonists were administered to identify the contribution of glutamate receptors to the firing of thoracic WDR neurons in SCI rats. The 2,3-dioxo-6-nitro-1,2,3,4-tetrahydrobenzo[f]quinoxaline-7-sulfonamide disodium salt (NBQX, 1 μg, *n* = 6, 6.4 ± 1.1 spikes/s, *p* = 0.016) and (5S,10R)-(+)-5-methyl-10,11-dihydro-5H-dibenzo[a,d]cyclohepten-5,10-imine maleate (MK-801, 50 μg, *n* = 6, 8.4 ± 1.6 spikes/s, *p* = 0.042) treatments significantly attenuated WDR neuronal firing compared to the SCI (*n* = 5, 16.2 ± 2.5 spikes/s) and vehicle (*n* = 6, 15.5 ± 2.4 spikes/s) groups (Figure 2C). 

### 2.3. Superoxide-Mediated Activation of CamKII 

**Figure 3:**
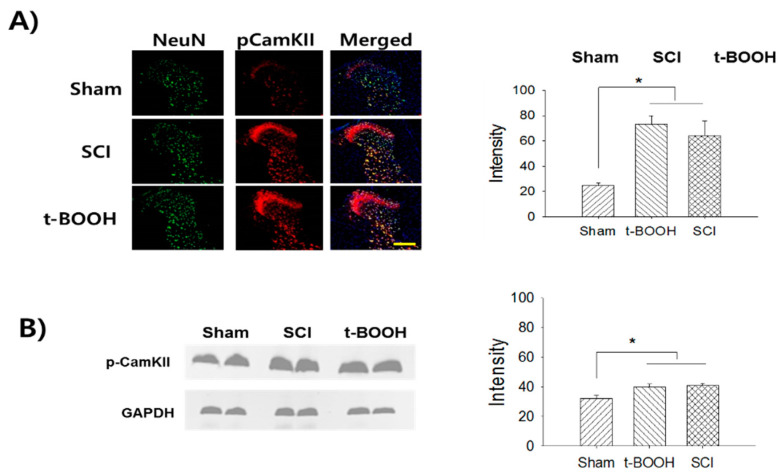
Superoxide-mediated activation of CamKII in the thoracic dorsal horn neurons. (**A**) The double immunofluorescence staining shows expression of pCamKII among the sham, SCI, and t-BOOH groups, respectively. The treatments of t-BOOH and SCI show an increase of pCamKII expression in the T7/8 thoracic dorsal horn neurons compared to the sham control group (* *p* < 0.05). (**B**) In Western Blot, t-BOOH and SCI groups show significant increases of pCamKII expression compared to the sham group. Scale bar: 300 μm.

The expression levels of pCamKII in the t-BOOH and SCI groups were 73.6 ± 4.5 (*p* < 0.001) and 64.3 ± 11.4 (*p* = 0.008), respectively, which were significantly higher than in the sham group (24.7 ± 1.9, Figure 3A). Western blot analysis confirmed that pCamKII expression in the t-BOOH (*p* = 0.037) and SCI (*p* = 0.031) groups increased significantly compared to the sham group (Figure 3B). 

### 2.4. Contribution of Superoxide and pCamKII to Mechanical Sensitivity in the Body Trunk

**Figure 4:**
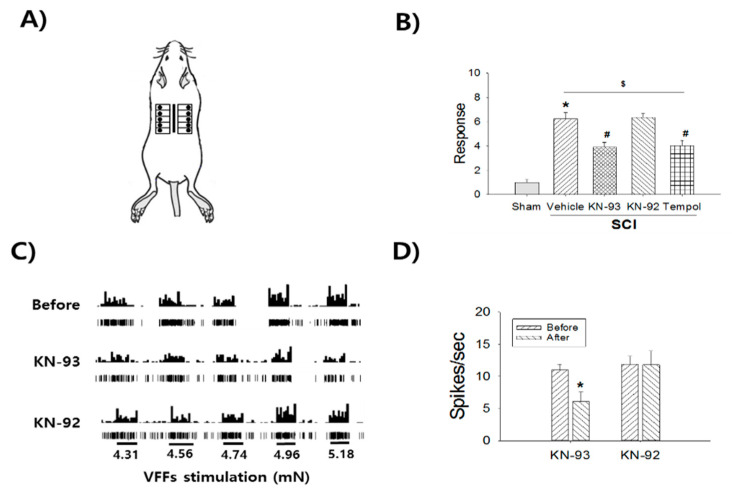
The contribution of pCamKII on mechanical sensitivity in the dorsal body trunk after SCI. (**A**) Dorsal trunks were divided by 10 square grids and 5.88 mN VFF was applied for 2 seconds at each square. Vertical bar: the incision of the back for the SCI surgery. Black circles: VFF stimulation dermatomes. (**B**) SCI (vehicle group) shows a significant increase of mechanical sensitivity compared to the sham group (* *p* < 0.05). However, KN-93 (50 μg/10 μL/kg) and Tempol (1 mg) treatments show significant decreases (^#^ *p* < 0.05), whereas KN-92 (50 μg/10 μL/kg) shows no significant changes compared to the vehicle group. However, all SCI groups show significant increases of mechanical sensitivity compared to the sham group (^$^ *p* < 0.05). (**C**) Histograms (upper) and waveforms (bottom) represent the changes of WDR neuronal firing rates. (**D**) KN-93 treatment shows significant decrease of neuronal firing rates at 30 min compared to the before treatment (* *p* < 0.05).

We then sought to determine whether inhibiting pCamKII activity and superoxide scavenging reduced mechanical sensitivity in the dorsal body trunk in the SCI-induced pain groups (Figure 4A). The mechanical sensitivity of the SCI + vehicle group (*n* = 7) increased significantly (6.2 ± 0.5) compared to the sham control group (*n* = 5, 1 ± 0.2, *p* < 0.001). The mechanical sensitivity after intrathecal administration of N-[2-[[[3-(4-chlorophenyl)-2-propenyl]methylamino]methyl]phenyl]-N-(2-hydroxyethyl)-4-methoxybenzenesulphonamide (KN-93, 50 μg, *n* = 9) was 3.9 ± 0.4, indicating significant attenuation compared to the SCI + vehicle group (6.3 ± 0.4, *p* = 0.003). In contrast, 2-[N-(4-methoxybenzenesulfonyl)]amino-N-(4-chlorocinnamyl)-N-methylbenzylamine (KN-92, 50 μg, *n* = 9) did not significantly attenuate mechanical sensitivity. The mechanical sensitivity after intrathecal administration of Tempol (1 mg, *n* = 9) was 4 ± 0.4, representing a significant decrease compared to the SCI + vehicle group (*p* = 0.005, Figure 4B). However, mechanical sensitivity was significantly different in all SCI groups compared to the sham group (*p* < 0.001, Figure 4B). To evaluate the contribution of pCamKII to the neuronal firing of WDR neurons, KN-93 (50 μg, *n* = 6) was applied on the spinal surface (Figure 4C). After a 30-min KN-93 treatment, the firing rate of WDR neurons was 6.1 ± 1.4 spikes/s, which represented a significant decrease compared to pre-treatment in the SCI group (11 ± 0.9 spikes/s, *p* = 0.047), whereas KN-92 treatment (50 μg, *n* = 4) did not cause a significant change (Figure 4D). 

### 2.5. Superoxide Production Following SCI

**Figure 5:**
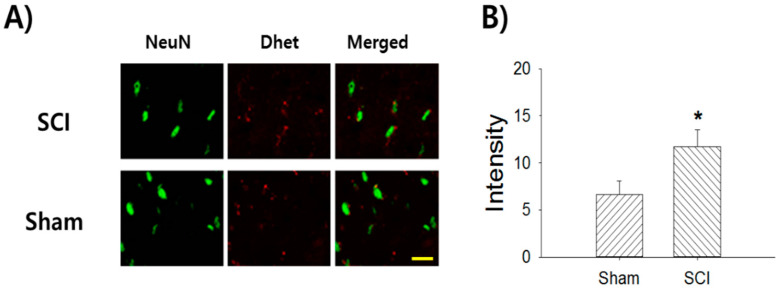
Increase of superoxide production in the thoracic T7/8 spinal dorsal horn after T10 SCI. (**A**) The immunofluoresence staining images represent the neuronal marker neuron (green), superoxide marker Dhet (red), and merged signal, respectively. (**B**) Rats with T10 thoracic spinal cord contusion injury show a significant increase of Dhet intensity in the T7/8 spinal dorsal horn compared to the sham control (* *p* < 0.05). Scale bar: 50 μm.

We examined whether SCI enhanced superoxide production in the at-level region of the spinal dorsal horn. Figure 5A shows the superoxide, as detected by dihydroethidium (Dhet; an autoimmunofluorescent agent that detects superoxide), in thoracic level T7/8 spinal dorsal horn neurons following a T10 contusion injury. Five weeks after SCI (7 rats), the mean intensity of Dhet in the SCI group was 11.7 ± 1.7, which represented a significant increase compared to the sham control group (*n* = 7 rats, 6.6 ± 1.5, *p* = 0.023, Figure 5B). 

## 3. Discussion

The present study suggests that the interaction between superoxide and CamKII contributes to neuronal hyperexcitability and at-level neuropathic pain following SCI. In addition, we suggest that the increase in superoxide triggers CamKII expression without the need for activation of ionotropic glutamate receptors. 

It is well known that removing ROS attenuates various pain conditions, such as peripheral neuropathic pain, bone pain, and inflammatory pain [17,18,19]. Herein, treatment with the ROS scavenger Tempol more rapidly inhibited neuronal discharge in response to mechanical stimulation compared to the PBN treatment. Tempol and PBN are ROS scavengers and easily cross the membrane due to their cell-permeable and hydrophobic properties [20,21,22]. Tempol is a stable compound, and acts as a scavenger of oxygen-derived radicals and as a nitric oxide spin trap. Although PBN is a non-specific ROS scavenger, it acts as a spin trap for short-lived radicals, such as nitrones [20]. The current study suggests that scavenging oxygen-derived radicals are more efficient at attenuating neuronal hyperexcitability and neuropathic pain. Previous studies have suggested that the interaction between superoxide and CamKII under neuropathic conditions is highly correlated with the signaling mechanisms in the spinal cord and brain [23,24,25,26]. The hydroxyl radical donor t-BOOH causes lipid peroxidation and mitochondrial calcium release via hydrolysis of pyridine nucleotide, which is a substrate of Ca^2+^-dependent signal transduction; this results in ROS-induced activation of CamKII [27]. In addition, a reduction in superoxide significantly decreases the mechanical hypersensitivity and CamKII-induced hyperexcitability of spinal WDR neurons in the spinal dorsal horn [28]. Taken together, these findings show that SCI-induced superoxide plays a key role in excitatory transmission and neuropathic pain after SCI. 

In the present study, the increase of superoxide was not strongly correlated with upregulation of ionotropic glutamate receptors, such as AMPA or NMDA, in the thoracic spinal dorsal horn. However, previous reports have shown that intrathecal treatment with AMPA/NMDA receptor antagonists inhibits WDR neuronal firing in SCI rats [15], and that acute intraspinal AMPA receptor blockade reduces ROS production and mitochondrial dysfunction [29]. In addition, an in vitro study revealed NMDA and AMPA receptor-mediated increases in intracellular ROS and calcium ions [30,31]. Previous studies have also reported roles of glutamate and its receptor in SCI-induced sensory and motor dysfunction. SCI-induced changes of glutamate showed spatial (dorsal horn vs. ventral horn) and temporal (acute (<24 h) vs. early stage (7 days)) differences [32,33,34]. However, the role of superoxide in ionotropic glutamate receptor and CamKII activation in SCI-induced chronic (>1 month) neuropathic pain is not well established. Therefore, we hypothesize that superoxide-induced at-level neuropathic pain is not directly mediated by the membrane-bound ionotropic glutamate receptor during the chronic phase after SCI. However, PBN treatment attenuated activation of AMPA/NMDA receptors and CamKII, as well as hyperalgesic behavior, in spinal lumbar dorsal horn neurons [18,35]. Taken together, the present study suggests that superoxide-mediated activation of the nociceptive signaling pathway and the ionotropic glutamate receptor will differ in temporal and spatial terms after SCI. 

**Figure 6:**
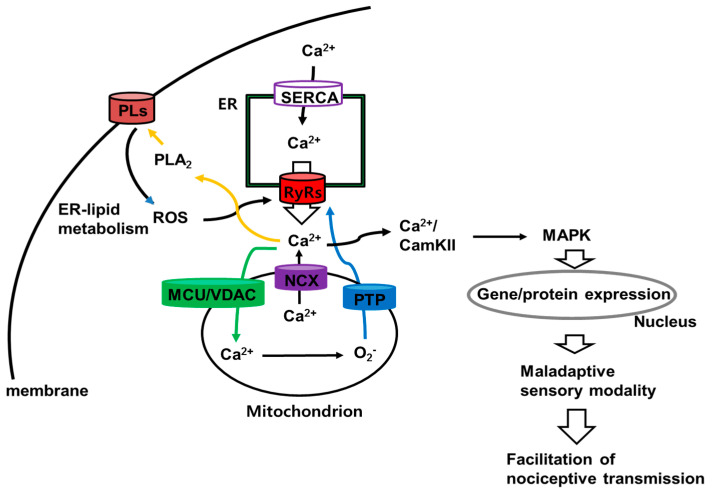
The schematic flow of ROS–CamKII interaction and facilitation of nociceptive transmission in SCI pain. The cytosolic calcium ions in ER are triggered by activation of sarco/endoplasmic reticulum Ca^2+^-ATP (SERCA). Activation of RyRs increase calcium ion efflux into cytoplasm and initiates calcium-dependent processes. (1) Calcium ions trigger the activation of calcium-dependent phospholipase A_2_ (PLA_2_) to cause the production of ROS via breakdown of membrane phospholipid (PLs, ER-lipid metabolism). (2) Following the activation of mitochondrial calcium uniporter/voltage-dependent anion channel (MCU/VDAC), mitochondrial calcium ions generate superoxide via mitochondrial respiratory chain complexes. Outflux of superoxide via permeability transition pore (PTP) in mitochondria activates ryanodine receptors (RyRs) in ER that result in increases of calcium ions outflux. (3) Mitochondrial calcium ions outflux via sodium/calcium exchanger (NCX) increase cytosolic calcium ions. (4) Calcium/CamKII-mediated activation of mitogen activates protein kinase family (p38MAPK, ERK, and Jun) that initiates the gene/protein expression, which is result in the facilitation of excitatory nociceptive transmission.

It is well known that an increase in calcium ion release from cytoplasmic calcium stores activates calcium-dependent protein kinase [36], and that activated CamKII maintains its active form throughout autophosphorylation [37,38,39]. These results suggest that ROS-mediated calcium levels are involved in the activation of CamKII, rather than the influx of calcium ions, which is mediated by the ionotropic glutamate receptor. Recent reports strongly suggest an interaction between ROS generation and calcium release from internal stores, such as the endo/sarcoplasmic reticulum (ER/SR) and mitochondria. For example, knockdown of the Ryanodine receptor (RyR) 2 in the SR prevents oxidative stress and mitochondrial dysfunction [40,41,42]. In addition, traumatic SCI causes RyR dysfunction due to oxidation/nitrosylation [43], while RyR inhibitors decrease mitochondrial superoxide generation and neuropathic pain [44,45]. The high sensitivity of RyRs to calcium ions, which are generally activated by a few micromolar of calcium, and their auto-regulatory activities (caused by calcium-induced calcium release) [46,47] enable RyRs to play a critical role in cytosolic calcium-mediated signaling, such as by activating the CamKII pathway. Those reports suggest that calcium ions play critical roles in intracellular excitatory nociceptive signaling events and the maintenance of chronic neuropathic pain. The literature demonstrates well that CamKII mediates the upregulation of extracellular signal-regulated kinases 1/2 and p38 kinases, which are a group of p38 MAPKs that contribute to at-level mechanical allodynia [48]. Taken together, these reports suggest that calcium ions are critically important in oxidative pathways and neuropathic pain following SCI (Figure 6).

The present study had some limitations as a pharmacological evaluation. First, we used only single doses of Tempol, PBN, and t-BOOH and did not induce motor failure, in accordance with previous reports [10,19,49]. In addition, the present study was concerned only with local spinal cord circuits, and the systemic effects of ROS were not examined. This can be justified based on our previous study indicating no significant differences in neuropathic pain attenuation between intrathecal and systemic administration of ROS [10]. A future study should perform a wide range of pharmacological tests to elucidate the role of ROS-mediated nociceptive signaling in SCI pain. 

In conclusion, our data suggest that the activation of CamKII signaling by superoxide contributes to at-level neuropathic pain without involvement of ionotropic glutamate receptors in the spinal dorsal horn.

## 4. Materials and Methods

### 4.1. Animals

Male Harlan Sprague Dawley (225–250 g, HyoChang Science, Seoul, Korea) rats were housed under a reversed 12 h/12 h light/dark cycle and fed ad libitum. To induce the thoracic contusion injury, the rats were anesthetized with an intraperitoneal (i.p.) injection of sodium pentobarbital (60 mg/kg). Following T8/9 dorsal vertebral laminectomy, the T10 spinal cord was contused by a force of 150 kdynes with a 1 s dwell time. Our previous study demonstrated that chronic neuropathic pain behaviors develop under these conditions [10,50]. The age-matched sham surgery was performed using the same procedure, except for the contusion injury. All injured rats were given daily injections of Baytril (enrofloxacin, 0.03%) for 5 days to prevent infection, and their bladders were expressed daily until they began to voluntarily void. All experimental procedures were reviewed and approved by the Animal Care Committee of Daegu Haany University (Korea, DHU2016-059, 28 June 2016) and carried out according to the National Institutes of Health Guide for the Care and Use of Laboratory Animals. 

### 4.2. Immunohistochemistry

To determine whether SCI leads to overproduction of superoxide in the injured thoracic spinal dorsal horn, Dhet (Invitrogen, Carlsbad, CA, USA) was administered intrathecally (50 μM) 24 h before perfusion. To detect ROS-mediated excitatory synaptic transmission, the expression levels of phosphorylated CamKII (p-CamKII), AMPA (GluR1 and GluR2/3), and NMDA (NR2A and NR2B) receptors were evaluated in the thoracic spinal dorsal horn using double immunofluorescence staining. Briefly, rats were deeply anesthetized with sodium pentobarbital (80 mg/kg, i.p.) and perfused intracardially with heparinized physiological saline followed by 4% ice-cold paraformaldehyde/0.1 M phosphate buffer (PB) solution. After perfusion, T7/8 was removed immediately and postfixed overnight in 4% paraformaldehyde/0.1 M PB, followed by cryoprotection in a 30% sucrose solution over several days. After fixation, the T7/8 spinal cords were individually embedded in optimal cutting temperature compound and sectioned to 20 μm thickness. Antibodies for NeuN (1:2000; Millipore, Burlington, MA, USA), GluR1 (1:300; Millipore), GluR2/3 (1:200; Millipore), NR2A (1:500; Millipore), NR2B (1:500; Millipore), and pCamKII (1:300; Santa Cruz Biotechnology, Santa Cruz, CA, USA) were incubated with a cocktail solution (0.1 M PB, 1% Triton X-100, and 3% normal goat serum) at room temperature for 1 day with gentle shaking. After four washes with 0.1 M PB, the sections were incubated with secondary antibodies for 2 h (1:600; Molecular Probes, Sunnyvale, CA, USA). The sections were collected by the free-floating method and mounted on gel-coated slides with mounting medium (DAPI). Two serial sections of the T7/8 dorsal horn area of each rat were randomly selected, captured by confocal microscopy, and evaluated by measuring the intensity.

### 4.3. Western Blot

To determine whether SCI changed pCamKII expression in the thoracic spinal dorsal horn, Western blotting was performed according to a previous report [24]. After administering pentobarbital (100 mg/kg), the rats were perfused intracardially with cold heparinized saline. Then, the T7/8 segments of the spinal cord were immediately removed, and the dorsal parts were dissected on dry ice. After homogenization, the homogenates were centrifuged at 10,000× *g* for 10 min. Protein concentrations were determined with a bicinchoninic acid (BCA) protein assay kit (Pierce, Rockford, IL, USA). After centrifuging the supernatant at 10,000× *g*, the samples were heated and loaded onto a polyacrylamide gel. The samples were separated by electrophoresis in Tris-glycine buffer at 300 V, and the proteins were transferred overnight to a polyvinylidene difluoride (PVDF) membrane at 30 V in transfer buffer. After blocking, the membranes were incubated overnight with the phosphorylated forms of the alpha subunit of CamKII (1:1000; Millipore). After washing, the membranes were incubated for 1 h in horseradish peroxidase-conjugated anti-rabbit IgG diluted 1:20,000 and washed three times. Peroxidase activity was detected by the ECL Plus detection system (Amersham, Piscataway NJ, USA), and images were collected by exposing the membranes on chemiluminescence film. The integrated density values of the signals were recorded.

### 4.4. Electrophysiology

An in vivo extracellular recording was made, using a single carbon filament (7 µm carbon fiber)-filled glass microelectrode (1–2 µΩ, Kation Scientific, Minneapolis, MN, USA) to measure the action potential firing rates of spinal T7/8 WDR dorsal horn neurons in response to VFF stimuli, according to a previous study [10]. Following anesthesia (sodium pentobarbital 60 mg/kg, i.p., supplemented by infusion of 5 mg/h/300 g via jugular vein cannulation), the rats were mounted in a stereotaxic frame apparatus. Body temperature was maintained at 37 °C and monitored with a rectal probe. WDR neurons were characterized by firing rates that increased with increasing intensity of VFF stimuli (4.31, 4.56, 4.74, 4.96, and 5.18 mN, Bioseb, Florida, USA) applied to the receptive field of the dorsal body trunk. The firing rates were combined and expressed as the rate per second. Single-cell activity was amplified by ISO-80 (low filter, 0.3 K; high filter, 3 K; gain, 10^4^) and processed using 1401 Plus system (Cambridge Electronic Design, Cambridge, UK). The real-time waveform and peristimulus histogram (spikes/1s bin width) were constructed with Spike 2 software (Cambridge Electronic Design, Cambridge, UK). The VFF stimuli were applied successively for 10 s each, with an inter-stimulation interval of 20 s. 

### 4.5. Mechanical Sensitivity at the Body Trunk

Mechanical sensitivity was measured at the dorsal dermatomes of the body trunk according to a previous study [48]. Briefly, a grid map (five bilateral points on the midline of the back) of the dorsal trunk was made and VFF stimuli were applied at a point lateral to the grid to avoid stimulation near the incision area. Mechanical sensitivity was determined based on avoidance, head turns, or vocalizations to VFF stimuli. For the VFF stimuli, log unit 5.88 mN rather than 5.18 mN was used, in accordance with a previous empirical study showing more robust responses to the former. The VFF stimuli were applied perpendicularly for 2 s (one in each grid square), and the average score for 10 applications was recorded. 

### 4.6. Drug Administration

To determine whether superoxide and pCamKII were involved in at-level pain behaviors, an intrathecal catheter (CS-1 Intrathecal Catheter; ReCathCo, Allison Park, PA, USA) was implanted from the cisterna magna to the T5/6 level, and 2 cm of the free end was left exposed at the nape of the neck to allow drug administration. The catheter was implanted 5 days before the test under inhalation anesthesia (isoflurane; induction, 3%; maintenance, 1.5%). Saline (20 μL) was injected daily to prevent clogging of the intrathecal catheter. After implantation, the rats were individually housed and monitored for secure implantation. The superoxide anion scavenger Tempol (1 mg/10 μL/kg; Sigma-Aldrich, St. Louis, MO, USA), hydroxyl radical donor t-BOOH (0.4 mg/10 μL/kg; Sigma-Aldrich), non-specific ROS scavenger PBN (3 mg/kg; Sigma-Aldrich), pCamKII inhibitor KN-93 (50 μg/10 μL/kg), and inactive enantiomer KN-92 (50 μg/10 μL/kg; EMD Biosciences, San Diego, CA, USA) were intrathecally administered, and the i.t. tubing was flushed with saline (10 μL). To determine the contribution of AMPA and NMDA receptors to the hyperexcitability of WDR neurons, we administered the AMPA receptor antagonist NBQX (1 μg) or NMDA receptor antagonist MK-801 (50 μg) to SCI rats. All drug doses were set according to previous studies [10,15,24].

### 4.7. Statistical Analysis

The behavioral data were analyzed using repeated-measures analysis of variance and the Student–Newman–Keuls test for multiple comparisons. Electrophysiological and immunohistochemical data were analyzed using the *t*-test. A *p* value < 0.05 was considered significant for all statistical tests, which were conducted using the SigmaPlot program (ver. 13.0; SPSS Inc., Chicago, IL, USA). Data are expressed as mean ± standard error.

## Data Availability

Not applicable.
